# Evaluation of the correlation between cerebral hemodynamics and blood pressure by comparative analysis of variation in cerebral blood flow in hypertensive versus normotensive individuals: A systematic review and meta-analysis

**DOI:** 10.17305/bb.2024.10230

**Published:** 2024-08-01

**Authors:** Lei Yang, Hong Du, Xuejing Zhang, Dongliang Zhang, Xianhui Su, Zongrong Qiao, Bulang Gao

**Affiliations:** 1 Department of Neurosurgery, Shijiazhuang People’s Hospital, Shijiazhuang, China; 2 Department of Cardiology, Second Hospital of Hebei Medical University, Shijiazhuang, China; 3 Center of Medical Research, Shijiazhuang People’s Hospital, Shijiazhuang, China

**Keywords:** Cerebral blood flow (CBF), hypertension, systolic blood pressure (SBP), diastolic blood pressure (DBP), normotension, cardiovascular risk factors, cerebral hemodynamic, systematic review, meta-analysis

## Abstract

Current understanding of the cerebral vascular response to variations in blood pressure (BP) among individuals with hypertension is limited. The aim of this meta-analysis was to determine the correlation between hypertension, risk of stroke, and cerebral blood flow (CBF). We reviewed studies published between 2000 and 2023 from PubMed, Google Scholar, and Science Direct that compared mean CBF in normotensive (NTN) and hypertensive (HTN) patients. A random effects model was used to construct the risk ratio (RR), 95% confidence interval (CI), forest plot, and inverse variance weighting. Additionally, a mixed-effects meta-regression was employed to examine the impact of study-specific patient variables. This meta-analysis included eight prospective cross-sectional studies published from 2002 to 2023. It revealed a significant average difference in the standard mean CBF of −0.45 (95% CI −0.60 to −0.30, *I*^2^ ═ 69%, *P* < 0.00001), distinguishing NTN from HTN subjects. A RR of 0.90 (95% CI 0.63 to 1.30, *I*^2^ ═ 89%, *P* ═ 0.04) indicated a significant decrease in CBF among individuals with hypertension. We found a statistically significant relationship between changes in diastolic and systolic BPs and the mean CBF (R ═ −0.81, *P* ═ 0.001 and R ═ −0.90, *P* ═ 0.005, respectively). Our research demonstrates a strong relationship between elevated BP and reduced CBF, with hypertension reducing CBF compared to NTN individuals, by increasing cerebrovascular resistance.

## Introduction

A persistent increase in blood pressure (BP) above the threshold of 130/90 mmHg is what distinguishes hypertension, also known as high BP. This leads to an excessive amount of force exerted by the blood on the arterial walls [1]. Hypertension exerts a significant impact on a substantial portion of the global population, as nearly 30% of the adult population between the ages of 30 and 79 are afflicted by this ailment [2]. The phenomenon of cerebrovascular remodeling, characterized by alterations in the morphology, structure of cerebral blood vessels, and changes in artery reactivity, such as impaired dilation, has been found to be associated with hypertension. The condition under consideration is distinguished by a reduction in the baseline cerebral blood flow (CBF) resulting from heightened rigidity in the peripheral blood vessels, including those located in the brain [3, 4]. Furthermore, it has been observed that individuals with hypertension demonstrate elevated levels of cerebrovascular resistance in comparison to normotensive (NTN) counterparts [5]. An increased susceptibility to cerebrovascular events, such as decreased blood flow due to stenosis, thrombosis, embolism, or hemorrhage, might be one of the reasons for the inability to regulate CBF [6, 7]. Inadequate circulation of blood, known as ischemia, can have deleterious effects on cerebral tissue, potentially leading to the onset of stroke and dementia [8, 9]. Research has shown that untreated hypertension, inadequate management of hypertension, and elevated BP levels are associated with a reduction in CBF [10, 11]. Furthermore, hypertensive (HTN) artery remodeling often causes a reduction in the diameter of the arterial lumen and an increase in the ratio of the arterial wall to the lumen in most cerebral arteries [12, 13]. Hypertension induces structural changes in cerebral blood vessels and interferes with complex vaso-regulatory processes that are responsible for maintaining sufficient blood flow to the brain. These modifications pose a potential risk to CBF. The regulation of cerebral circulation is primarily governed by alterations in vascular resistance. Modulation of resistance can occur through the influence of local-chemical and endothelial factors, as well as autacoids. Additionally, nonvascular control, or functional hyperemia, is the physiological process where increased neuronal activity leads to a corresponding increase in regional CBF. This ensures an adequate delivery of oxygen and nutrients to the activated brain region. Hypertension has been observed to reduce resting CBF, alter the intrinsic innervations crucial for neurovascular coupling, and influence endothelial-dependent responses. Additionally, most cerebral arteries undergo HTN arterial remodeling, resulting in a reduction in lumen diameter and an increase in the wall-to-lumen ratio. It is widely believed that these modifications contribute to a decrease in mean CBF following ischemia and an increase in ischemic damage [14, 15]. Currently available information regarding the cerebral vascular response to fluctuations in BP in individuals with hypertension remains limited. The objective of this systematic review and meta-analysis was to examine the existing literature [16–23] pertaining to the relationship between hypertension and CBF, aiming to establish a correlation between these two variables.

## Materials and methods

The meta-analysis was carried out in accordance with the recommendations provided by the Preferred Reporting Items of Systematic Reviews and Meta-Analyses (PRISMA) [24].

### Data sources and search strategy

This meta-analysis was conducted following a comprehensive search across multiple databases, including PubMed, Embase, Scopus, and the Cochrane Library. Between 2000 and 2023, the search employed precise terms, including “cerebral blood flow” OR “CBF,” “systolic blood pressure” OR “SBP,” “diastolic blood pressure” OR “DBP,” “cerebral hemodynamics” OR “CHD,” “cardiovascular risk factors” OR “CVD,” “meta-analysis,” “cross-sectional studies,” and “systematic review.” Using the PICO framework [25], terms that were consistent in both Medline and EMBASE databases were identified. The Title (ti)-Abstract (abs)-Keyword (key) field was used to search Scopus with the provided keywords. The terms “cerebral blood flow velocity” and “hypertension” were employed in the Cochrane database.

The PICO structure was employed to formulate specific selection criteria [25]. In this particular context, the letter “P” represents individuals with hypertension, the letter “I” refers to the variability in the velocity of blood flow in the brain, the letter “C” represents individuals with normal BP, and the letter “O” encompasses several clinical outcomes, including systolic blood pressure (SBP), diastolic blood pressure (DBP), and the average blood flow in the brain. The design methodology used in this study was limited to the use of (A) prospective or retrospective studies that reported the average CBF in NTN and HTN groups, (B) studies that included patients with hypertension who were over 18 years old, and (C) studies that provided the main outcome data: SBP, DBP, and average CBF in both HTN and NTN groups. The inclusion criteria specified that only papers published in the English language were considered. The selection of papers was performed following the guidelines of PRISMA. Two researchers, referred to as XZ and DZ, individually conducted an extensive examination of the relevant literature to identify pertinent studies.

### Study selection

The search for the relevant literature was carried out and the following requirements had to be fulfilled for a study to be considered eligible: Studies that are cross-sectional and evaluate the mean CBF in both NTN and HTN groups, and studies that evaluate the primary outcomes, which include SBP, DBP, and mean CBF in both HTN and NTN groups. Studies that reported hypertension-induced changes in the hemodynamics of pregnant women were among the studies that were excluded from consideration. In addition, studies that were carried out on healthy volunteers or those who suffered from conditions other than hypertension were not included in this meta-analysis.

### Data extraction

Microsoft Excel was used to create a computerized data extraction form, which was then utilized for documenting the fundamental information of the studies that were chosen for this meta-analysis. The name of the first author, the year of publication, the journal of publication, the type of study, the total number of participants, the number of individuals with hypertension and those with non-hypertension, the mean age of patients, the gender ratio (male to female), the cardiovascular parameters studied, the cerebral hemodynamic parameter studied, and the instrument that was used for the analysis were all included. Extraction of the data was carried out independently by two distinct writers, and the outcomes of the extractions carried out by both authors were subsequently compared. Despite the fact that there were contrasting viewpoints, a consensus was reached through conversation. A third author was also featured in the work, but this was contingent upon the circumstances.

**Figure 1.**
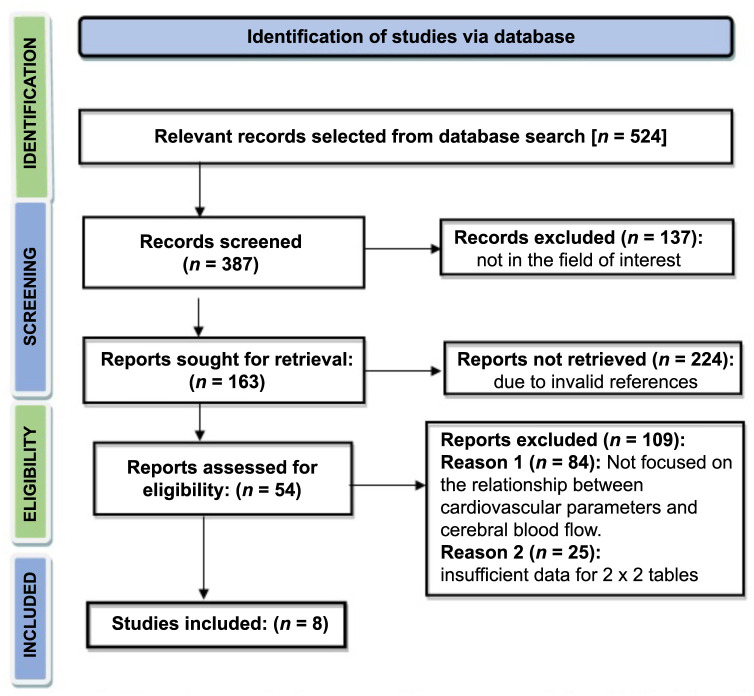
**PRISMA flowchart for the selection of studies**. PRISMA: Preferred Reporting Items of Systematic Reviews and Meta-Analyses.

### Quality assessment

The Cochrane Risk of Bias tool, which is published in version 5.3 of the Cochrane Handbook, was applied in order to evaluate the methodological validity of each study that was included in the meta-analysis [26]. Selected articles were given a rating of “low,” “high,” or “some concern” during the process of data extraction. This rating was determined by the methodology of the articles, which included a variety of factors such as the generation of random sequences, the concealment of allocation, the blinding of participants and staff, the blinding of outcome assessment, the insufficiency of outcome data, selective reporting, and potential sources of bias. This information was taken into consideration when constructing a risk of bias summary and graph for evaluating the quality of the work. XZ and DZ, two different evaluators, participated in an independent investigation into the possibility of bias. Additionally, XS, who was recognized as an additional reviewer, acted as an arbitrator to settle any issues that remained unresolved. After this, the funnel plot [27] was applied to determine whether or not publication bias was present, and the MedCalc program [28] was utilized to carry out Begg’s test [29] in order to determine whether or not it was statistically significant.

### Ethical statement

Since this study relies solely on publicly available published literature, it was not required to obtain authorization from a clinical ethics committee.

### Statistical analysis

Through the utilization of RevMan software version 5.4 and the implementation of a meta-analysis, the average change in CBF (mL blood/100 mL tissue min) was obtained across all of the investigations. It was determined that a random effects model with inverse variance weighting was used to estimate the mean change that occurred after surgery. This model took into account the predicted heterogeneity. The findings were displayed in the form of a forest plot, which also included the confidence interval (CI) for the 95% level of certainty. In order to evaluate the influence of particular parameters, such as SBP, DBP, and mean change in CBF, across a number of different studies, a mixed-effects meta-regression [30] was utilized in the review process. To investigate the influence of each variable on the outcomes of various research studies, a model was constructed that incorporated either the mean (standard deviation) or the frequency of each variable in each study. This investigation used data collected at the study level and included the application of meta-regression. In addition, box and whisker plots [31], correlation plots [32], and scatter plots [33] were generated in order to visualize the associations between the variables.

## Results

### Literature search results

Figure 1 shows the PRISMA chart for the selection of research. Through a comprehensive search of online databases, 524 studies were identified and 137 studies were excluded due to irrelevant fields. Following a review of the title and abstract, 387 papers were then examined. Subsequently, 163 reports were sought for retrieval, following the elimination of 224 reports deemed irrelevant due to factors such as absence from valid databases, publication in languages other than English, or classification as articles from online encyclopedias or blogs. Out of these, 109 papers were further eliminated; 84 lacked data on cardiovascular risk factors and CBF, and 25 lacked sufficient information to construct a 2 × 2 table. Eight cross-sectional studies that were eventually relevant were finally included in the current meta-analysis after 54 reports were first evaluated for eligibility. Table 1 displays the characteristics of each individual included in the chosen research. Included studies assessed how high BP affected mean CBF.

**Table 1:** Characteristics of the included studies

**Study ID**	**Year of publication**	**Journal of publication**	**Type of study**	**Total number of participants**	**Hypertensive patients**	**Normotensive patients**	**Patients using antihypertensive medications**	**Age of patients, mean ± SD**	**Sex (M/F)**	**Cardiovascular parameters**	**Cerebral hemodynamic parameters**	**Instrument used**
De Ciuceis et al. [16]	2014	Neuroradiology	Cross-sectional study	20	10	10	Yes	HTN: 65 ± 12 NTN: 62 ± 11	HTN: 8/2 NTN: 7/3	SBP, DBP	CBF	Contrast MRI
Dai et al. [17]	2008	Stroke AHA	Cross-sectional study	41	19	22	Yes	HTN: 82.6 ± 3.6 NTN: 82.2 ± 3.7	HTN: 7/12 NTN: 7/15	SBP, DBP	CBF	Continuous arterial spin-labeled MRI
Fitri et al. [18]	2020	Journal of Hypertension	Cross-sectional study	330	160	170	Yes	HTN: 62.4 ± 8.1 NTN: 62 ± 8	HTN: 7/12 NTN: 7/15	SBP, DBP	CBF	TCD
Jennings et al. [19]	2006	Neurology	Cross-sectional study	96	37	59	No	HTN: 61.3 NTN: 60	HTN: 7/12 NTN:12/25	SBP, DBP	CBF	Phase contrast MRI
Machado et al. [20]	2020	Journal of Clinical Hypertension	Cross-sectional study	115	85	30	Yes	HTN: 43.42 ± 11 NTN: 50.57 ± 11.7	HTN: 18/12 NTN:32/	SBP, DBP	CBF	Phase contrast MRI
Neumann et al. [21]	2019	Hypertension AHA	Cross-sectional study	39	13	26	Yes	HTN: 57.6 ± 9.7 NTN: 52.9 ± 8.9	HTN:12/14 NTN: 43/42	SBP, DBP	CBF	Phase contrast MRI
Serrador et al. [22]	2004	Journal of Applied Physiology	Cross-sectional study	60	22	38	Yes	HTN: 72 ± 5 NTN: 70 ± 4	HTN:12/14 NTN: 43/42	SBP, DBP	CBF	TCD
Traon et al. [23]	2002	Journal of the Neurological Sciences	Cross-sectional study	42	21	21		HTN: 48.9 ± 13.6 NTN: 51 ± 14.5	HTN: 13/8 NTN: 13/8	SBP, DBP	CBF	TCD

SBP: Systolic blood pressure; DBP: Diastolic blood pressure; CBF: Cerebral blood flow; MRI: Magnetic resonance imaging; TCD: Transcranial doppler; HTN: Hypertensive patients; NTN: Normotensive patients.

### Risk of bias assessment and publication bias

**Figure 2.**
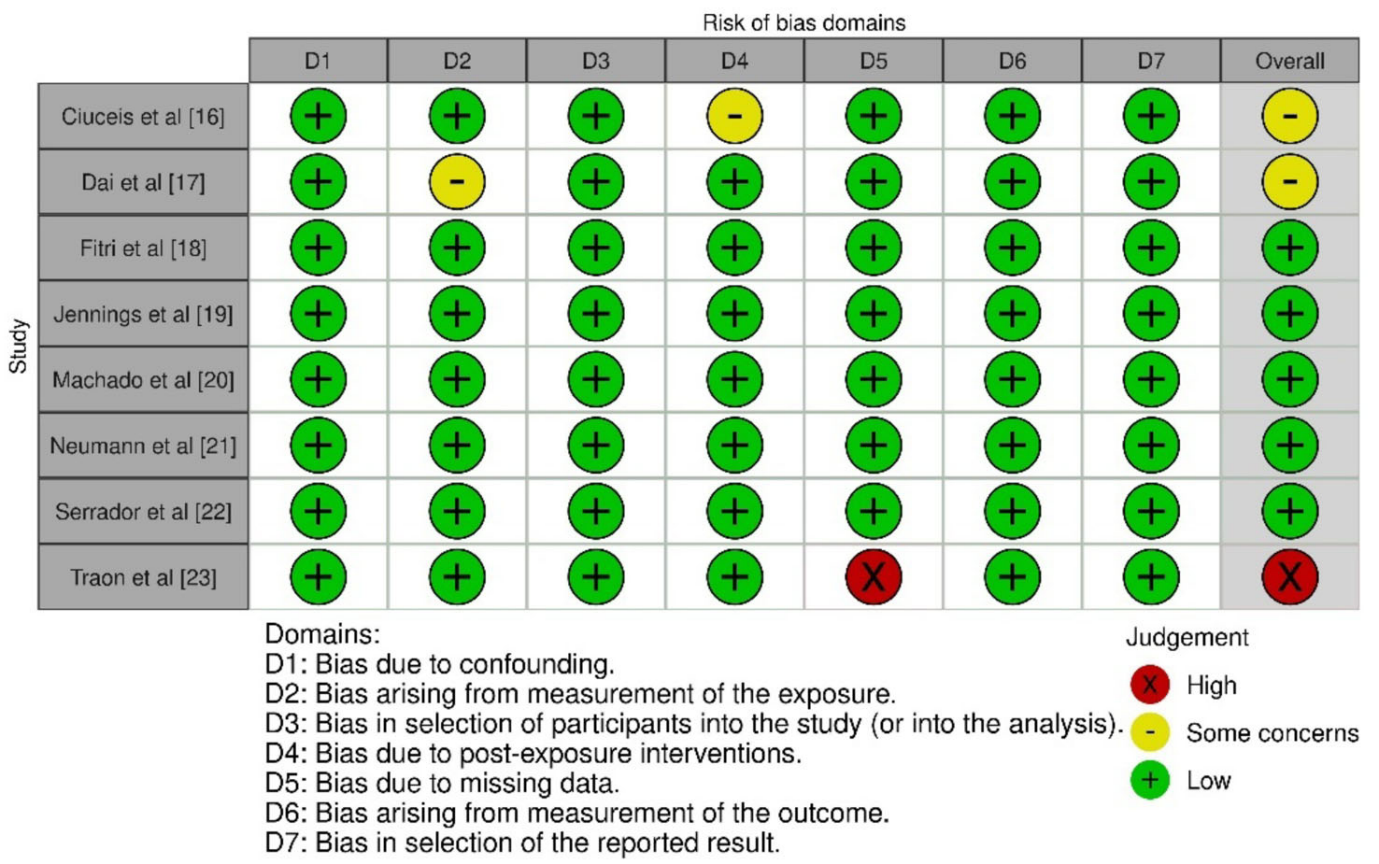
**Risk of bias summary of the included studies**.

**Figure 3.**
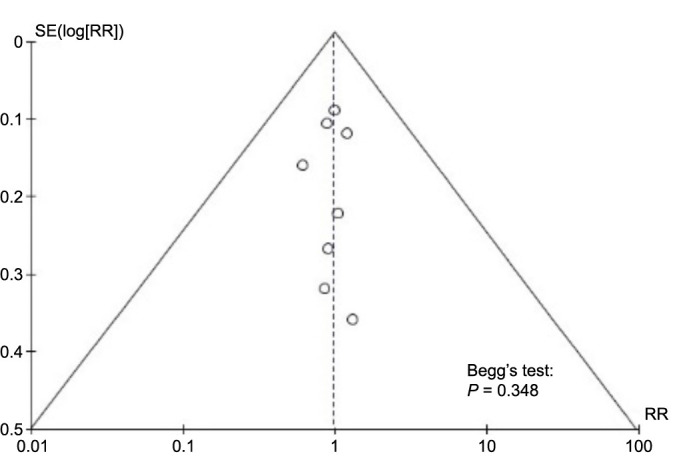
**Funnel plot for publication bias.** SE: Standard error; RR: Risk ratio.

A summary of the potential for bias is presented in Figure 2, which also includes an evaluation of the quality of the studies that were included. A low risk of bias was found in six of the eight studies that were included in the analysis. On the other hand, two of the studies had a moderate risk of bias due to bias that was caused by the assessment of the exposure and bias that was caused by post-exposure treatments. There was a strong potential for bias in one of the included studies since there was a lack of data. The graph depicting the potential for bias can be found in Figure S1. Figure 3 represents the funnel plot, which revealed that there was a low chance of publication bias, as demonstrated by a *P* value of 0.348 for Begg’s test [34], which was not significant.

### Meta-analysis results

**Figure 4.**
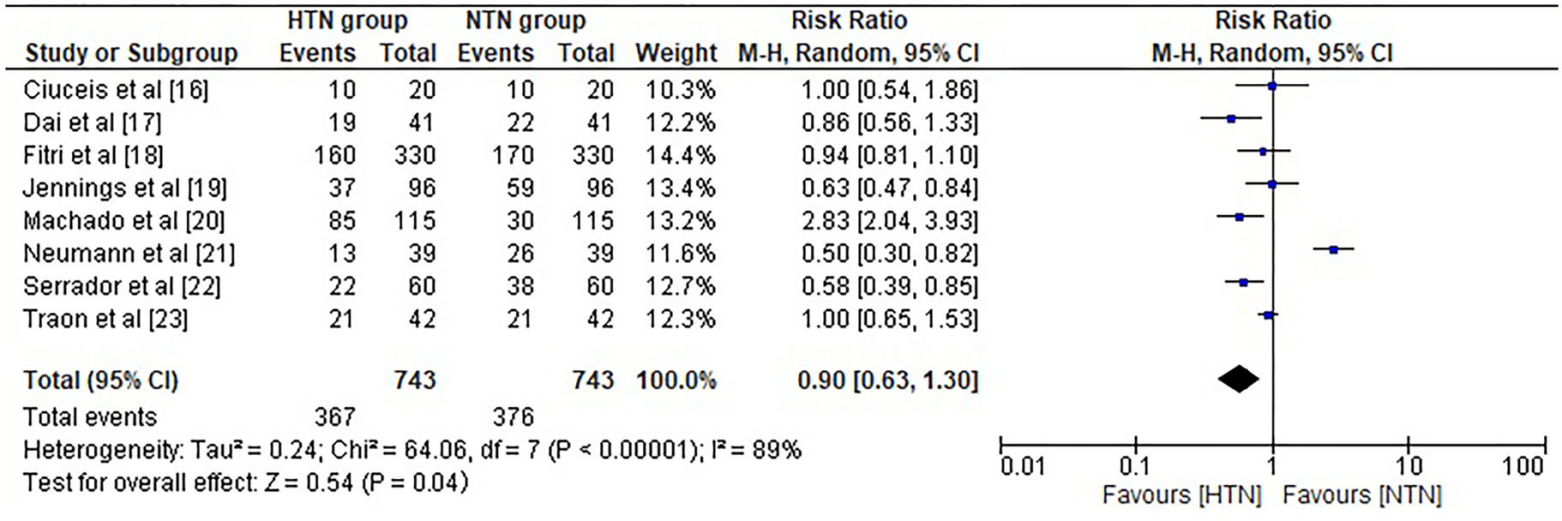
**Forest plot for risk ratio of reduction in mean cerebral blood flow in hypertensive vs normotensive group**. CI: Confidence interval; HTN: Hypertensive patients; NTN: Normotensive patients.

**Figure 5.**
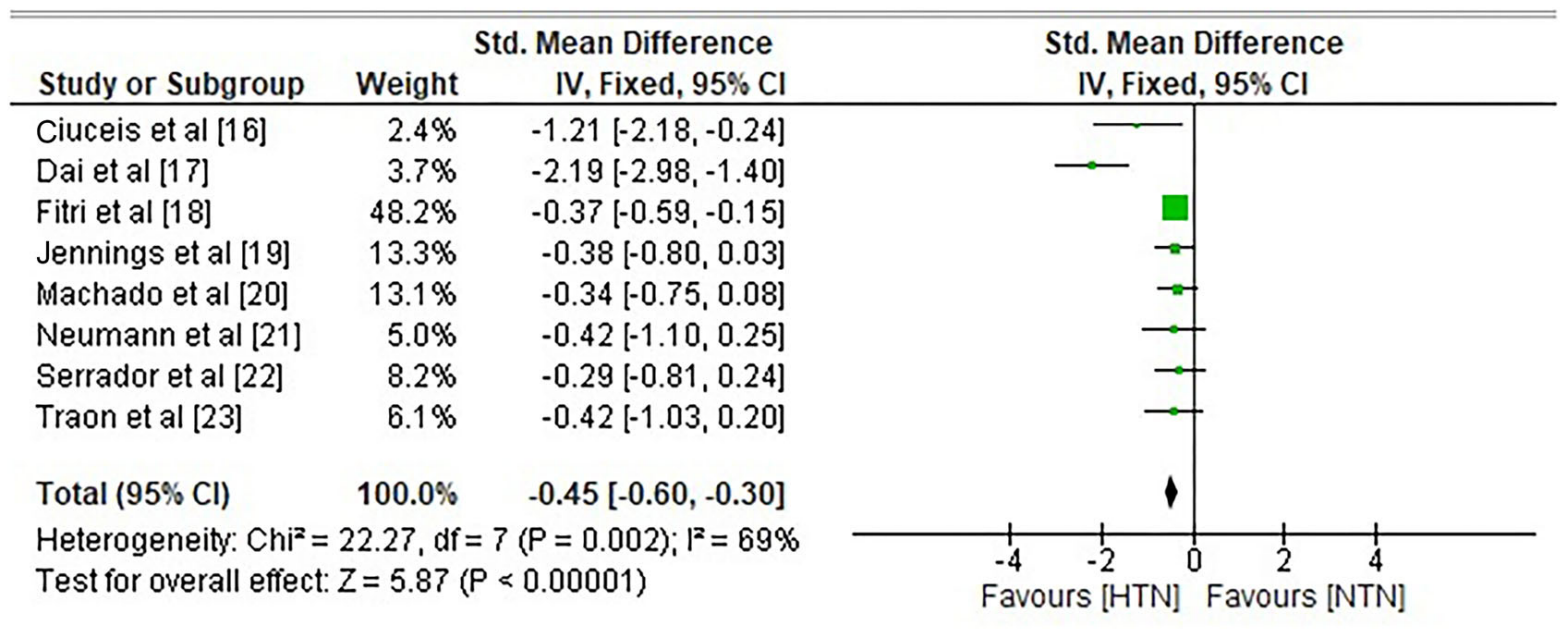
**Forest plot for variation in mean cerebral blood flow in hypertensive vs normotensive group**. CI: Confidence interval; HTN: Hypertensive patients; NTN: Normotensive patients.

The risk ratio (RR) or relative risk, evaluates the risk of a reduction in CBF in the hypertension group compared to the risk of the same event in the NTN group. It was found that HTN individuals showed a significant reduction in CBF with a RR of 0.90 (95% CI 0.63–1.30) and substantial heterogeneity of Tau^2^ ═ 0.24, Chi^2^ ═ 64.06, df ═ 7, *I*^2^ 89%, *Z* ═ 0.54, *P* ═ 0.04 (Figure 4). All the included studies demonstrated a statistically significant difference in mean CBF between NTN and HTN individuals with a standard mean difference of −0.45 (95 % CI −0.60 to −0.30) and heterogeneity of Chi^2^ ═ 22.27, df ═7, *I*^2^ ═ 69%, *Z* ═ 5.87 and *P* < 0.00001 as shown in Figure 5.

### Across study findings (meta-regression)

**Figure 6.**
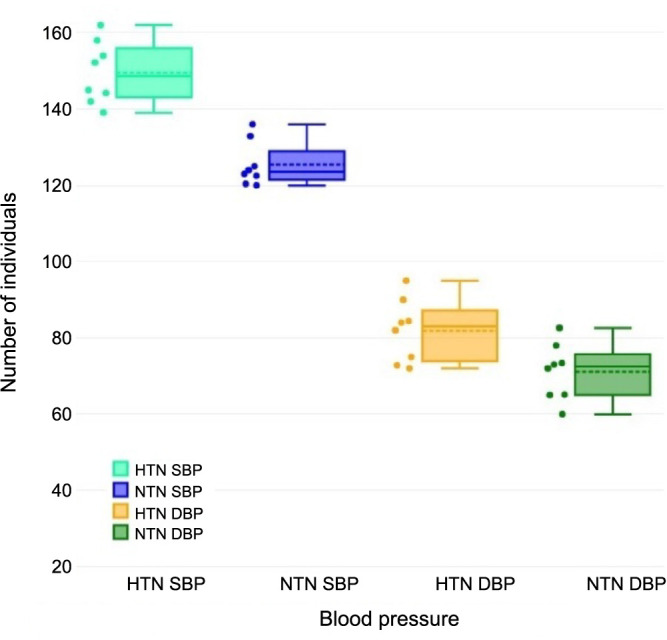
**Box and whisker plots for comparative cardiovascular parameters in hypertensive vs normotensive group**. HTN: Hypertensive patients; NTN: Normotensive patients; SBP: Systolic blood pressure; DBP: Diastolic blood pressure.

The study employed univariate meta-regression analysis to examine the association between hypertension and the mean CBF. Figure 6 presents the box and whisker plot, which provides the five-number summary [35]. For the SBP of the HTN group, min ═ 139.1, Q1 ═ 143.1, median ═ 148.6, Q3 ═ 156, and max ═ 162 with a mean value of 149.56. For the SBP of the NTN group, min ═ 120, Q1 ═ 121.45, median ═ 123.5, Q3 ═ 128.95, and max ═ 136 with a mean value of 125.47. For the DBP of the HTN group, min ═ 72, Q1 ═ 73.9, median ═ 83, Q3 ═ 87.2, and max ═ 95, with a mean value of 81.9. For the DBP of the NTN group, min ═ 60, Q1 ═ 65.05, median ═ 72.5, Q3 ═ 75.7, and max ═ 82.6, with a mean value of 71.13. For the mean CBF of the HTN group, min ═ 25, Q1 ═ 30.5, median ═ 43, Q3 ═ 55.5, and max ═ 58 with a mean value of 42.64. Finally, for the mean CBF of the NTN group, min ═ 32, Q1 ═ 39.5, median ═ 46.5, Q3 ═ 60.19, and max ═ 63 with a mean value of 48.4. All six groups have a potentially symmetrical skew and a mesokurtic tail. This summary reveals a substantial correlation between the extracted data pertaining to the primary cardiovascular factor, specifically SBP, DBP, and the mean CBF, which was analyzed across the included studies (Table 2).

**Table 2:** Summary of the primary study parameters

**Study ID**	**Cardiovascular parameters**				**Cerebral hemodynamic parameters**	
	**Systolic blood pressure**		**Diastolic blood pressure**		**Mean CBF**	
	**NTN**	**HTN**	**NTN**	**HTN**	**NTN**	**HTN**
De Ciuceis et al. [16]	123 ± 7	142 ± 16	73 ± 9	82 ± 10	57.59 ± 3.14	54.15 ± 2.23
Dai et al. [17]	120.0 ± 18.0	139.1 ± 20.8	65.1 ± 9	72.8 ± 10.4	44.0 ± 9.6	25.0 ± 7.0
Fitri et al. [18]	124 ± 7	145 ± 6	65 ± 5	72 ± 8	32 ± 9	29 ± 7
Jennings et al. [19]	120.4	144.2	73.4	84.4	46 ± 6	43 ± 10.0
Machado et al. [20]	122.5 ± 11	158 ± 6.6	60 ± 10.51	75 ± 13.2	62.8 ± 11.7	58 ± 15
Neumann et al. [21]	132.9 ± 15.3	152.2 ± 15.1	82.6 ± 8.7	90 ± 6.4	47 ± 10.7	43 ± 4.9
Serrador et al. [22]	125 ± 11	162 ± 7	72 ± 6	84 ± 7	35 ± 11	32 ± 9
Traon et al. [23]	136 ± 15	154 ± 16	78 ± 13	95 ± 11	63 ± 16	57 ± 12

Values represent mean ± SD. CBF: Cerebral blood flow; HTN: Hypertensive patients; NTN: Normotensive patients.

**Figure 7.**
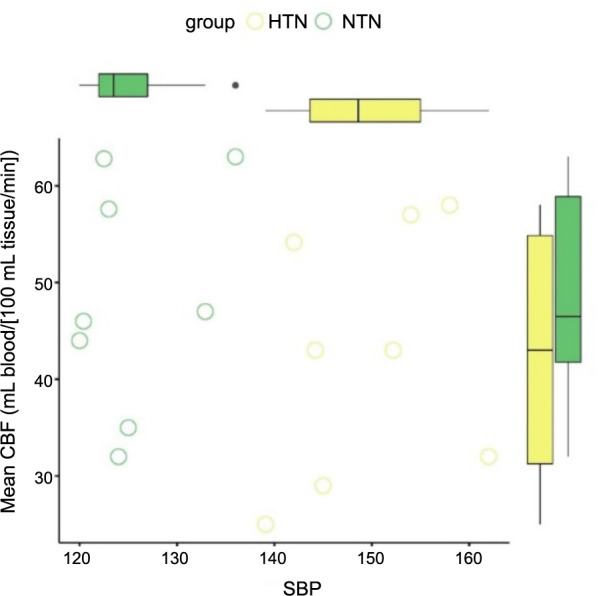
**Correlation plot for mean cerebral blood flow and systolic blood pressure in hypertensive vs normotensive group**. CBF: Cerebral blood flow; HTN: Hypertensive patients; NTN: Normotensive patients; SBP: Systolic blood pressure.

**Figure 8.**
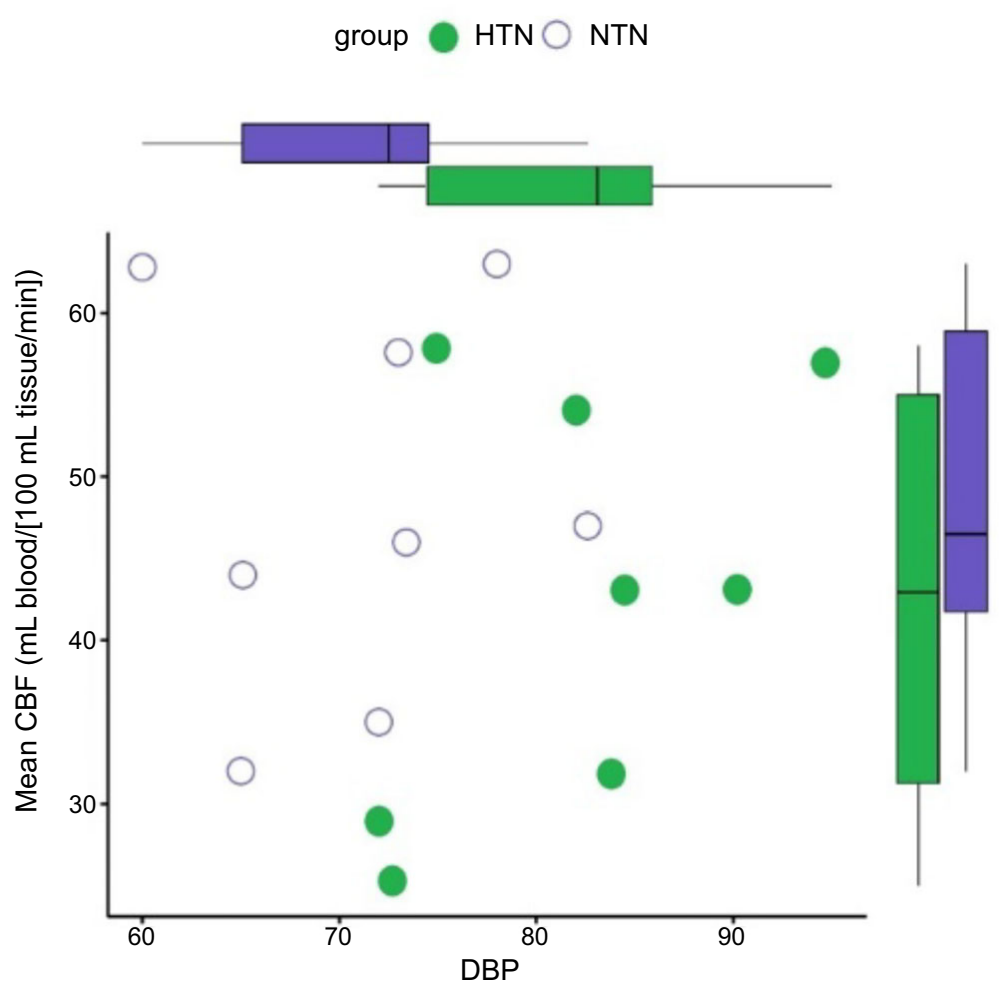
**Correlation plot for mean cerebral blood flow anddiastolic blood pressure in hypertensive vs normotensive group**. CBF: Cerebral blood flow; HTN: Hypertensive patients; NTN: Normotensive patients; DBP: Diastolic blood pressure.

**Figure 9.**
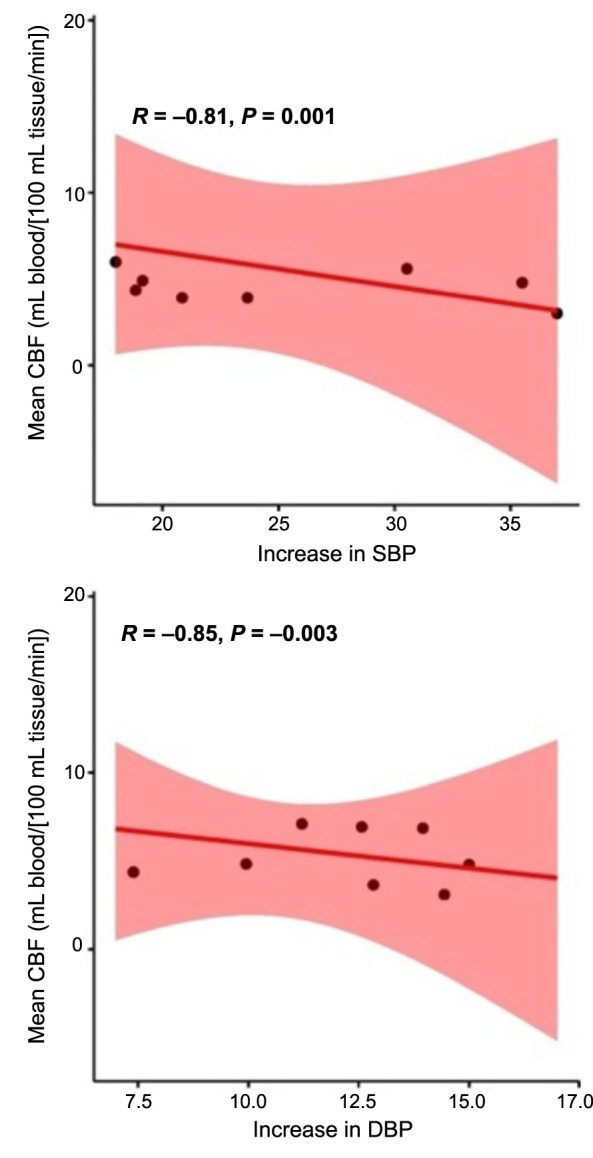
**Scatter plot for mean CBF and change in blood pressure.** Increase in (A) systolic blood pressure and (B) diastolic blood pressure in hypertensive vs normotensive group. CBF: Cerebral blood flow; SBP: Systolic blood pressure; DBP: Diastolic blood pressure.

**Figure 10.**
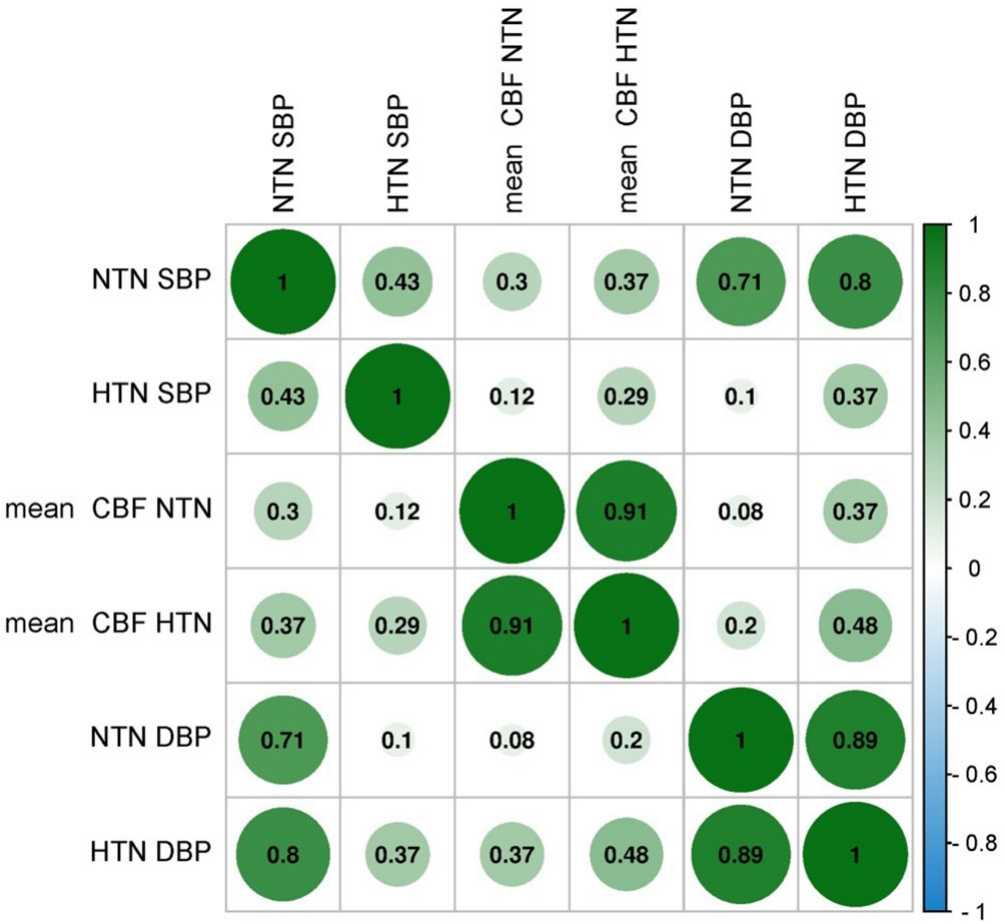
**Correlation plot for cardiovascular risk factor with cerebral hemodynamics for mean cerebral blood flow, systolic blood pressure and diastolic blood pressure**. HTN: Hypertensive patients; NTN: Normotensive patients; SBP: Systolic blood pressure; DBP: Diastolic blood pressure.

The findings shown in Figure 7 demonstrate a statistically significant association between the mean CBF and BP in both the HTN and NTN groups. The box and whisker plot demonstrates a decrease in the mean CBF value within the HTN group, which can be attributed to a notable increase in SBP. In a similar vein, the box and whisker plot presented in Figure 8 illustrates the correlation between the mean CBF and DBP in the HTN group. The plot reveals a noteworthy decrease in mean CBF, which can be attributed to a substantial increase in DBP. The scatter plots [36] shown in Figure 9 demonstrate a negative correlation between the average CBF and the changes observed in both SBP (R ═ −0.81, *P* ═ 0.001) and DBP (R ═ −0.90, *P* ═ 0.005). These findings further support the notion that an increase in BP is associated with a decrease in mean CBF. The robustness of the correlation is validated by the prominent correlation coefficient plot [37] shown in Figure 10.

## Discussion

The presence of hypertension is linked to cerebrovascular remodeling, a decrease in baseline CBF, and elevated cerebrovascular resistance compared to individuals without hypertension [38]. The brain possesses the capacity for autoregulation of CBF, enabling it to maintain a relatively stable blood flow even in the face of fluctuations in BP. Consequently, a deficiency in the ability to regulate CBF is closely associated with an elevated vulnerability to cerebrovascular events and the onset of dementia [39, 40]. The fundamental issue that has to be addressed with regard to CBF in the setting of hypertension is the disruption that is seen in the homeostasis of CBF. To be more specific, an increase in cerebrovascular resistance causes an upward migration of both the lower and upper thresholds of CBF self-stabilization to higher pressure values [41–43]. The fundamental process seems to include the structural thickening of cerebral resistance arteries as well as the luminal constriction of these veins [44]. According to a number of studies, the adaptive changes that were discussed previously and that were designed to protect the brain from having high intravascular pressure also contributed to enhancing the brain’s susceptibility to ischemia in conditions when the BP was low [45].

**Figure 11.**
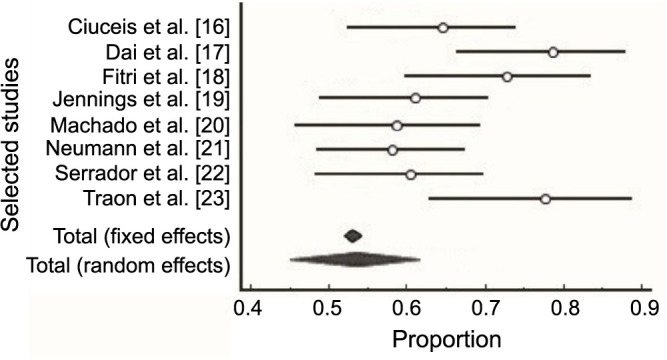
**Meta analysis plot for proportion of included studies**.

Eight studies were included in this meta-analysis, which examined the differences in CBF dynamics between NTN and HTN patients. Each study made a substantial contribution to this meta-analysis (Figure 11).

De Ciuceis et al. (2014) [16] utilized dynamic susceptibility-weighted contrast magnetic resonance imaging (DSC-MRI) in a selected cohort to investigate CBF and the morphology of the cerebral small-resistance arteries in individuals with normotension and hypertension. They removed cerebral small-resistance arteries from a very small segment of morphologically normal cerebral tissue and placed them on an isometric myograph in order to determine the media-to-lumen (M/L) ratio. This was done in order to carry out the evaluation. A region-of-interest method was then utilized in order to do a quantitative analysis of the CBF and cerebral blood volume (CBV). The researchers discovered that HTN patients exhibited a significantly lower regional CBF (mL/100 g/min) in the following regions: The cortical gray matter (mean ± SD: 55.63 ± 1.90 vs 58.37 ± 2.19, *P* < 0.05), the basal ganglia (53.34 ± 4.39 vs 58.22 ± 4.45, *P* < 0.05), the thalami (50.65 ± 3.23 vs 57.56 ± 4.45, *P* < 0.05), the subcortical white matter (19.32 ± 2.54 vs 22.24 ± 1.9, *P* < 0.05), the greater M/L ratio (0.099 ± 0.013 vs 0.085 ± 0.012, *P* < 0.05), and the lower microvessel density (1.66 ± 0.67 vs 2.52 ± 1.28, *P* < 0.05). It was observed that there was a statistically significant negative association between the M/L ratio of cerebral arteries and CBF in the cortical grey matter (r ═ −0.516, *P* < 0.05), basal ganglia (r ═ −0.521, *P* < 0.05), thalami (r ═ −0.527, *P* < 0.05), and subcortical white matter (r ═ −0.612, *P* < 0.01).

Dai et al. (2008) [17] conducted a study in which they investigated the abnormal regional CBF (rCBF) in cognitively normal elderly subjects who were also suffering from hypertension. In order to determine rCBF at 1.5 T, continuous arterial spin-labeled magnetic resonance imaging (MRI) was utilized, along with the deformable atrophy-corrected registration method. Researchers found that patients with hypertension who were cognitively normal had decreased recurrent CBF in the putamen, bilateral globus pallidus, and left hippocampus when compared to patients with normotension. In addition, it was discovered that the recurrent CBF was lower in the inferior parietal, left orbitofrontal, and left superior temporal cortices. Additionally, it was found to be lower in the left and right anterior cingulate gyrus with broadening to the subcallosal region, the left posterior cingulate gyrus, and the medial precuneus, as well as the left lateral inferior and superior frontal regions. A comparison of the CBF in the posterior circulation of patients with NTN and HTN BP was carried out by Fitri and Batubara (2020) [18] using transcranial direct current (TCD) examination. The researchers discovered that HTN patients had significantly lower mean flow velocities in the right vertebral artery (RVA) (29.71 ± 7.97 vs 32.74 ± 9.07, *P* ═ 0.001) and the left vertebral artery (LVA) (29.71 ± 9.68 vs 32.61 ± 9.06, *P* ═ 0.005). Additionally, the LVA had a lower peak systolic velocity at 50.57 ± 13.9 vs 54.11 ± 14.5, *P* ═ 0.024, and the RVA had a peak systolic velocity at 48.3 ± 15.30 vs 52.6 ± 15.01, *P* ═ 0.011.

Jennings et al. (2005) [19] utilized carotid artery ultrasound and MRI evaluations to examine compensation and decreased CBF response in patients with untreated hypertension. It was discovered that HTN subjects who exhibited a moderate level of proficiency in verbal memory demonstrated an increased rCBF response in the right amygdala or hippocampus. Additionally, HTN individuals have a diminished rCBF response in the parietal cortex. Using digital plethysmography and TCD, Machado et al. (2020) [20] assessed cerebral autoregulation performance in patients with arterial hypertension undergoing drug treatment. They observed that NTN individuals had a lower resistance-area product (1.17 ± 0.24, *P* < 0.05) in comparison to those with uncontrolled hypertension. Neumann et al. (2019) [21] examined the CBF low response to simulated hypovolemia in individuals with essential hypertension using phase-contrast MR angiography. The researchers discovered a significant drop in CBF and cardiac output during lower body negative pressure (LBNP), with a *P* value of less than 0.0001. The heart rate exhibited an increase during LBNP, with a significant rise observed at a pressure of -50 mmHg (*P* < 0.0001). The mean arterial BP remained constant during LBNP, with no significant change observed (*P* ═ 0.3). Serrador et al. (2005) [22] examined the relationship between cerebral pressure and blood flow in people with hypertension using transcranial Doppler. The researchers discovered that individuals with high BP showed an improved reduction of fluctuations in CBF when their BP changed, both at the cardiac frequency (lower gain) and in the low-frequency region (autoregulatory, 0.03–0.07 Hz). Although HTN individuals had an improved autoregulatory response to pressure, they showed less responsiveness to carbon monoxide.

The dynamics of CBF autoregulation in HTN patients were explored by Traon et al. (2002) [23] using TCD in the middle cerebral artery. Following this, the cerebral vascular resistance index (CR) was calculated. It was shown that the CR slope, which is an indicator of the rate of cerebral autoregulation, was comparable in both groups and among the HTN patients. This was the case regardless of whether the patients’ BP was adequately controlled (eight patients) or not controlled (13 patients). In both the control group and the HTN group, the amount of time it took for the CR to reach its greatest reduction (T1) and the amount of time it took for the CR to fully recover after the first dip (T2) were comparable. T1 recorded 11.3+/−3.1 s, while T2 recorded 12+/−5.9 s in the group that served as the control. The value of T1 in the HTN group was 11.7+/−2.5 s, and the value of T2 was 10.7+/−4.5 s. These timeframes were likewise constant among the patients who were diagnosed with hypertension.

One of the clear implications of the alteration in the lower threshold of CBF homeostasis in individuals who have hypertension is that when the BP of an HTN patient is suddenly reduced to “normal” levels, it falls below the patient’s lower autoregulatory threshold, which could be associated with ischemic injury [46]. Some studies showed a preserved ability to regulate flow compared with NTN individuals [47, 48], while other studies showed an impairment of regulation due to the fact that hypertension was associated with structural changes in response to increased transmural pressure, such as arterial stiffening and atherosclerosis [49–51]. The results of these studies showed that the regulation of CBF in hypertension in response to changes in BP shows mixed results. Patients with hypertension had the greatest decrease in CBF between the initial visit and the 4-year follow-up, as shown by Muller et al. [52] in large-scale longitudinal research involving 575 patients with evident vascular illness. The investigation was conducted over the course of four years.

In the present meta-analysis, we also found a statistically significant difference in mean CBF between NTN and HTN individuals with a mean difference of −5.47 (95 % CI −8.47 to −2.47; *I*^2^ 80%, *P* ═ 0.0004) and a higher likelihood of a reduction in CBF in HTN individuals with an RR of 0.90 (95% CI 0.63–1.30, *I*^2^ 89%, *P* ═ 0.04). Since the CBF was evaluated using MRI at varying Tesla levels in some studies and via TCD, in other studies, this variation results in a significant level of analysis heterogeneity. We were able to obtain a significant negative correlation between mean CBF and change in SBP (R ═ −0.81, *P* ═ 0.001) and DBP (R ═ −0.90, *P* ═ 0.005), which validated the prominent correlation between hypertension and dynamics of mean CBF.

### Limitations

In the context of this study, there are a number of constraints that need to be taken into consideration. Due to the small number of research, precisely eight, which display moderate to high degrees of variability, the conclusions are limited in their scope. The analysis was conducted in accordance with the established norms of scientific rigor, which is an important point to note. Second, rather than using the data from each individual participant, we used the data that was compiled from the entire research project in our analysis. Third, the sex of the patient was a significant determinant in relation to BP and CBF. Furthermore, there was some variation in the criteria used to select HTN patients and the methods employed to measure the CBF across the different studies, potentially introducing a certain level of internal heterogeneity. This situation persisted as the trials exhibited minor variations among them. Furthermore, it is worth noting that there were slight variations in the specific description of primary outcomes among the numerous studies included in the analysis. Additionally, it is important to acknowledge that the search conducted for this study only included publications written in English, potentially introducing bias in the selection of papers for inclusion.

## Conclusion

The findings of our research reveal a significant correlation between hypertension and CBF. Hypertension is characterized by cerebrovascular remodeling and a decrease in CBF relative to NTN individuals, primarily due to heightened cerebrovascular resistance. Hence, the regulation of BP is crucial in managing cardiovascular risk, as it plays a significant role in maintaining optimal cerebral hemodynamics. Deficiencies in CBF have been associated with cognitive decline and an increased susceptibility to ischemia.

## Supplemental data

**Figure S1.**
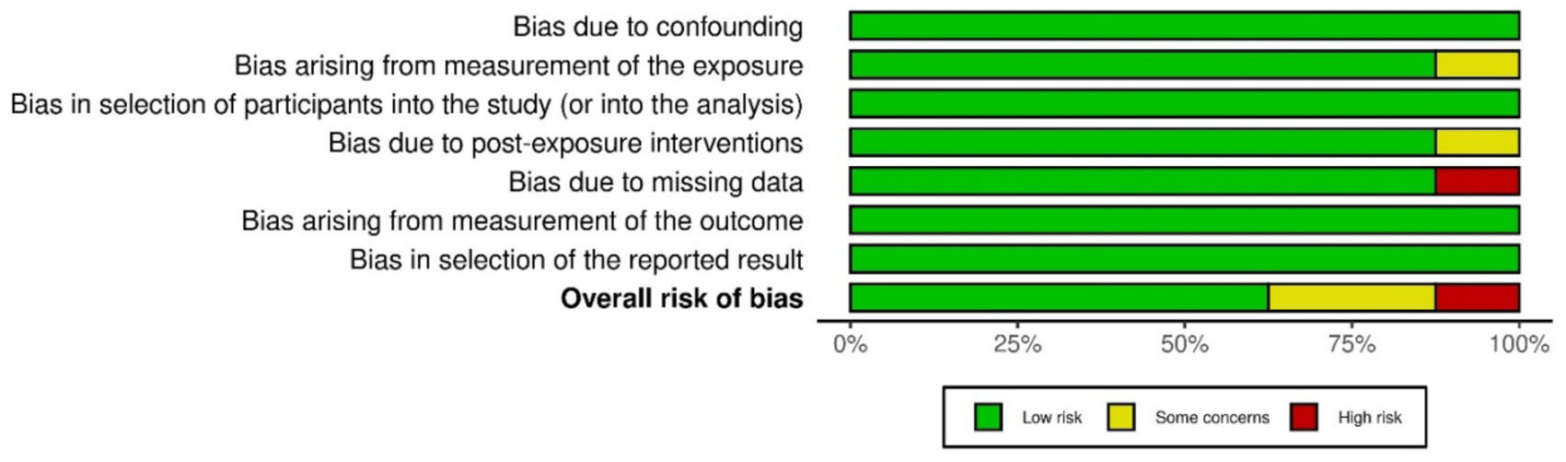
Summary plot for assessment of risk of bias.

## Data Availability

Upon reasonable request, the corresponding author will provide access to the requested information.
